# Influence of Lateral Cephalometric Radiographs on Orthodontic Treatment Planning of Class II Patients

**DOI:** 10.2174/1874210601812010296

**Published:** 2018-04-16

**Authors:** Irina Stupar, Enver Yetkiner, Daniel Wiedemeier, Thomas Attin, Rengin Attin

**Affiliations:** 1 Private Practice, Schulzahnklinik Nord, Hofwiesenstrasse 379, 8050 Zürich, Switzerland; 2 Faculty of Dentistry, Ege University, Bornova 35100 Izmir, Turkey; 3 Department of Statistics, University of Zurich, Zürich, Switzerland; 4 Department of Preventive, Periodontology, Cariology University of Zurich, Zürich, Switzerland; 5 Department of Orthodontics, Faculty of Dentistry, University of Zurich, Zürich, Switzerland

**Keywords:** Cephalometric radiographs, Orthodontic treatment, Class II patients, Tooth extraction, Linear Likert-type scale, Dento-alveolar malformations

## Abstract

**Background:**

Lateral Cephalometric Radiographs (LCR) are a common decision-making aid in orthodontic treatment planning and are routinely used in clinical practice. The aim of this present study was to test the null hypothesis that LCR evaluation does not alter specific components of orthodontic treatment planning in Class II patients.

**Materials and Methods:**

Records of 75 patients, who had been treated at the Department of Orthodontics, Centre of Dental Medicine, University of Zurich comprised the study material. Inclusion criteria were: (1) adolescents between the age of 12-15, (2) permanent dentition with Class II buccal segment relationship (3) absence of craniofacial and dento-alveolar malformations. Fifteen orthodontists from the dental faculties of Istanbul University, Istanbul and Ege University, Izmir filled out Likert-type linear scale questionnaires without knowing that they would repeat the same procedure with and without LCRs at two different time points. Equivalence and clinical relevance were assessed using (%95 CI) Wilcoxon signed rank tests.

**Results:**

Extraction decision did not differ between groups (*p*=0.68). Preference of functional appliance use (*p*=0.006) and inter-maxillary fixed functional appliance (p=0.043) was different among groups.

**Conclusion:**

LCR evaluation has minor influence on treatment planning procedure of Class II patients. It might be beneficial to consider its prescription not in a routine manner but as a supplementary tool considering possible reduction of radiation exposure.

## 1 INTRODUCTION

Orthodontic treatment planning is usually based on detailed subjective information obtained from the patient and objective diagnostic records (clinical examination, photograph evaluation, cast analysis and radiographs), which are evaluated by an orthodontist [1, 2].

Of all diagnostic means, radiographs and their routine prescription remains a critical issue as to proven harms of radiation [3, 4]. Especially mentioned is the Lateral Cephalometric Radiography (LCR), which is considered as the “gold standard” at the beginning of an orthodontic treatment [1, 5, 6]. An average number of three lateral cephalometric radiographs was reported to be taken during an orthodontic treatment [7].

Even though the contemporary radiographic innovations in dental medicine relatively reduced radiation exposure [8, 9], especially with the help of digital imaging and processing [10], the harmful effects of radiation are not justified unless it has the potential of changing one’s diagnostic decision [10, 11]. Since the majority of orthodontic patients are children and adolescents, the risk of ionizing radiation accumulation during their lifetime is higher than adults [12].

LCR is a two-dimensional diagnostic tool for identifying growth patterns, dentofacial proportions and relations between skeletal and dental structures, pathologies and occlusal discrepancies [1, 2]. It does not offer information about the transverse cranial level.

Since Silling *et al* [13] scrutinized the actual need of LCR in orthodontic treatment planning; several other studies asked the same question [4-6, 11, 14]. LCR seemed to have a higher impact on diagnosis than on treatment planning [6]. On the contrary, it was reported that clinical examination and dental casts might be sufficiently informative to estimate future skeletal development under certain circumstances [6, 15, 16].

Tooth extraction, one of the most invasive interventions in orthodontics to generate extra space, is usually supported by findings from the LCR analyses deviating from average norms [5, 11]. Moreover, it was reported that orthodontists might have personal tendencies for extraction or non-extraction therapies [5, 9], making the decision inter alia through LCR.

These observations raise the question, if the presence of LCR is influential on orthodontic treatment decisions, especially on irreversible decisions such as extractions.

Therefore, the aim of this study was to investigate the influence of LCR on orthodontic treatment planning in Class II patients at two points of time (T1 & T2) with or without LCR. The null hypothesis was that the use of LCR does not influence the treatment planning stage of Class II patients.

## 2 MATERIALS AND METHODS

### 2.1 Study Design and Subjects

**Fig. (1):**
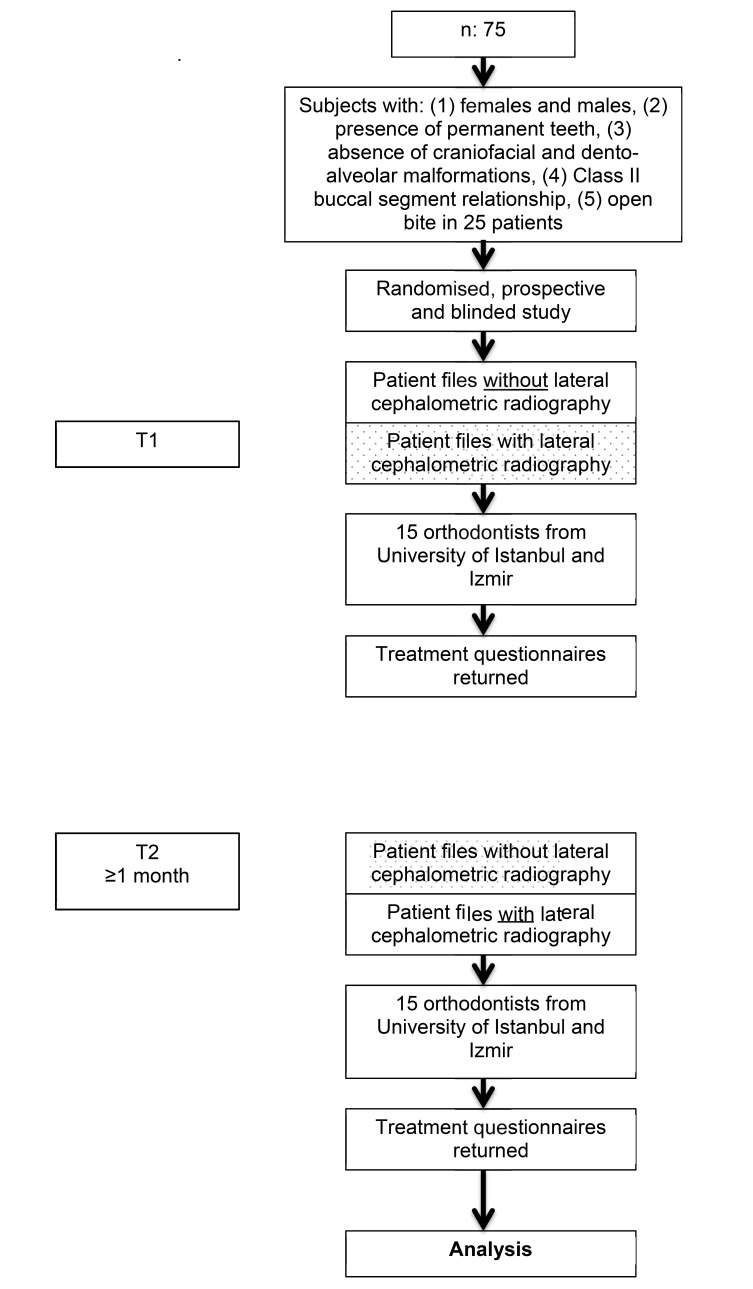
Flowchart of the study design.

Five orthodontists from Ege University, Izmir and ten orthodontists from Istanbul University participated the study as evaluators. They were not informed about the aim or subject of the study (Fig. **1**). Complete pre-treatment diagnostic files of seventy-five Class II patients from Department of Orthodontics and Pedodontics, University of Zurich archive were collected. These subjects fulfilled the following criteria: (1) permanent dentition (2) absence of craniofacial and dento-alveolar malformations, (3) Class II buccal segment relationship.

Files contained dental casts including cast analysis results, extra-oral photographs, panoramic radiographs and lateral cephalograms with associated tracings. Patient files were anonymized and numbered. Pictures (en-face, profile & ¾ profile) were masked. All data were digitally presented and there was no time limit for evaluating the cases and decision-making. The principal treatment objective was to accomplish a healthy functional occlusion with soft tissue harmony [11]. There was no restriction given in materials or financial conditions for treatment planning [11].

Half of the patient files did have LCR with the analysis, the other half were without LCRs. After four weeks (T2) the procedure was repeated with the same set of patient records excluding the LCR analysis of the patients, who had at T1 the complete radiographic analysis (LCR), and *vice versa* (Fig. **1**).

The questionnaire was designed as a linear Likert-type scale corresponding to a previous study [9]. With regard to the question of extraction or non-extraction, following questions had to be answered for each case: (1) definitely extraction, (2) extraction, (3) borderline, may or may not extract, (4) non-extraction, (5) definitely non-extraction.

Additionally, other ten therapy possibilities were also evaluated: (1) removable appliance, restricted to one jaw, (2) lower lingual arch and/or transpalatal arch, (3) headgear or skeletal anchorage, (4) functional appliance, (5) combinations of multiple choices 3 and 1, 2 or 3, (6) 3. Or 4. followed by fixed appliance, (7) only fixed appliance, (8) intermaxillary fixed functional, (9) extraction, (10) surgical treatment, (11) retention: a. fixed retention, b. removable retention, c. functional retainer.

The discrepancy of questions at T1 and T2 were observed and calculated (T2-T1). The bigger the discrepancy, the more likely the answers differed from each other. Those differences were visually displayed in Fig. (**2**).

### 2.2 Statistical Analysis

The ordinal scale data was encoded in Excel and statistically analyzed with the software R and plots were done with the ggplot 2 package.

Due to the cross-over study design and the ordinal scale of the target variables, a Wilcoxon signed-rank-test was used to determine significant differences between the treatment (with LRC) and control (without LRC) groups.

Equivalence and clinical relevance were assessed by considering the 95%-Confidence Interval (CI) of the Wilcoxon signed rank tests. Significant results were considered at *p* < 0.05.

## 3 RESULTS

Figs. (**2** and **3**) show a graphical overview regarding eleven analyzed choices, which were tested on respectively significant differences. Exclusion of LCR did not make a difference in any of the questions.

Decision of extraction *vs.* non-extraction therapy was indifferent (*p*=0.68). The power of 0.08 was low, supporting the assumption that there was no difference between control and orthodontic decision making with LCR.

Every single point illustrates the discrepancy of answers of each orthodontist to each question (T2-T1). For example: The answer for question 1 at T1 was definitely yes equal to 1, but at T2 it was definitely no, which corresponds to 5, from this it follows that the discrepancy is 4 (T2-T1), which is marked as a single point at the y-axis.

In Fig. (**2**) the discrepancy per question is shown. 0 in the y-axis stands for no differences between with or without LCR. Points are slightly jittered in order to improve the visual assessment. There is no asymmetrical pattern recognizable, in other words the deviation is balanced on both sides without any tendency. Every point means one discrepancy of one orthodontist. The bigger the difference, the more likely the answers differed from each other (T2-T1). All differences were non significant (*p*>0.05) except for question four, concerning functional appliance (*p*=0.006) and question eight, intermaxillary fixed functional appliance (*p*=0.043).

The y-axis represents the percentage of differences in answering each question. It is visible that over 50% of answers did not differ at T1 and T2.

## 4 DISCUSSION

In this study, the influence of LCR on different treatment decisions in Class II patients was tested. No evidence of a difference between treatment planning with or without LCR was found. Therefore, the null hypothesis that the cephalometric evaluation of Class II patients would not affect the treatment planning stage cannot be rejected.

The number of evaluators has a potential for possible bias, however, the focus was on verifying a difference between specific treatment decisions with/without LCR, neglecting possible individual factors of each orthodontist. Therefore, it can be assumed that 15 evaluators from two different centers might resemble an average population of orthodontists. The patient files were presented digitally and this might be an influencing factor on the orthodontists’ decisions since they are used to evaluating patients physically in real life and this might have affected the reliability of data. Similarly, dental casts were on photographs and not physically presented, which might have impeded the evaluation. However, it was shown previously that two-dimensional digital images can be used as an alternative to study casts to examine the actual need of an orthodontic treatment [15] and this was the only way of presenting the patient files to visualize the patient in professional platforms and discussions [11].

Exclusion of the LCR did not influence the orthodontic treatment decision of extraction, which is rather an irreversible decision. This recognition corresponds with previous studies [6, 11, 14, 16]. The contribution of the LCR to orthodontic decision making, as one of the essential orthodontic diagnostic materials might be questionable [5, 6]. LCR was considered as a gold standard in previous years since the main aim of orthodontic treatment was to treat the patient to cephalometric norms. With the paradigm shift of orthodontic aim from cephalometric norms to treating the face to harmonious soft and hard tissue relations, contribution of this tool gradually became questionable. Clinical evaluation of individual static and dynamic components started to become more decisive rather than average cephalometric values. Therefore, additional use of LCR remains controversial due to the individual character of each orthodontic treatment plan [13-16]. Considering the routine clinical inspection and the following evaluation of the diagnostic materials consisting of dental casts, photographs and panoramic x-rays, LCR might not be essential unless providing supplementary information in certain cases. Thus, routine prescription may be unnecessary exposure to radiation. Previously, it has been suggested that information set of radiography at initial treatment and obtain initial information out of study casts alone [15], would definitely reduce unnecessary radiation doses and even increase the benefit out of LCR [17]. The good agreement between stages of orthodontic therapy means, either on clinical examination or on the base of study casts was already described [14, 16].

The shortcomings of traditional radiographic imaging are another subject to discuss in terms of justification of ionizing radiation. Even though the trend of digital radiography could reduce the amount of ionizing radiation, the biological risk for growing individuals, as the biggest segment of orthodontic patient population, remains [8-10]. The danger of low-dosage radiation in children is still fully not clarified [9, 10]. As it is anticipated, each radiographic image has its own sources of drawbacks like magnification-distortion or positioning errors, which may increase the amount of ionization [1, 11, 18].

A recent study [11] reported a possible tendency to extraction with/without LCR in the level of experience of the individual orthodontist according to dichotomized results. The inconsistency in the results was interpreted as a disparity in field experience [11]. This difference in years of experience in correlation to the use of cephalographs might be an objective for a further study and would answer the question, whether the presence or non-presence of LCR is differently influencing experienced and inexperienced clinicians.

LCR is used routinely for supporting the orthodontic decision to extract or non-extract, an irreversible and invasive treatment [4, 5, 19-21]. Teeth extraction does not only influence the facial appearance regarding soft tissues and dental arch length dimensions [2, 5], but also presents a psychological impact on the patient. Baumrind and coworkers [5] stated regarding the extraction decision, orthodontists were even more focused on appearance related factors, which were visible on dental casts and facial photographs, than on radiography [5]. Even if some may criticize the precision of analyzing skeletal orthodontic issues without radiographic help, it was noted that clinicians could simply distinguish a Class I profile when compared to Class II and Class III by only clinical examination [22].

In the present study, the extraction of third molars was excluded, which might apply as a disadvantage, because of the possible participation in a treatment planning regarding the posterior dental arch. The exclusion of third molars into decision prevented potential positive results of extraction. One other limitation was the limited number of evaluators and centers. Information gathered from a higher number of orthodontists from a larger group of centers would represent the orthodontic community more accurately.

## CONCLUSION

The presence of LCR does not influence the orthodontic decision to extract or non-extract. Thus, the need of LCR in Class II patients should be reconsidered, additionally to prevent unnecessary ionizing radiation and reassess the routinely use in orthodontics.

## Figures and Tables

**Fig. (2):**
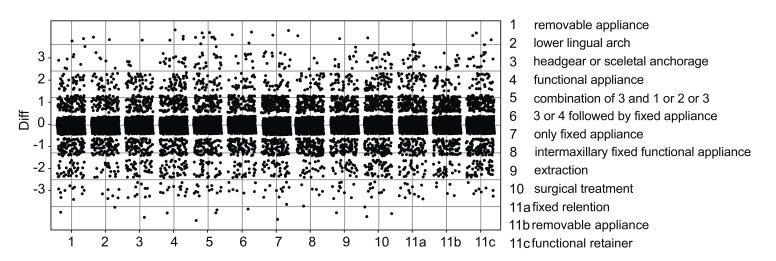
Graphical overview. Every single point illustrates the discrepancy of answers of each orthodontist to each question (T2-T1). For example: The answer for question 1 at T1 was definitely yes equal to 1, but at T2 it was definitely no, which corresponds to 5 – from this it follows that the discrepancy is 4 (T2-T1), which is marked as a single point at the y-axis.

**Fig. (3):**
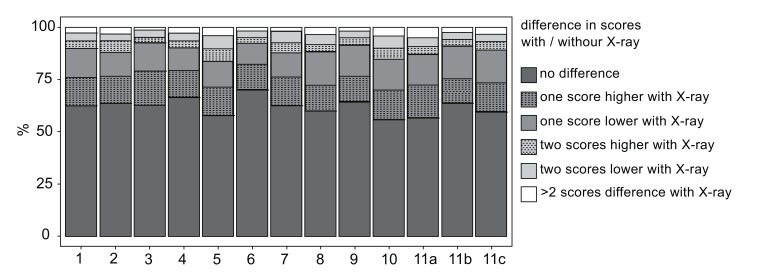
Difference in scores with / without X-ray. All examined questions are listed in the x-axis from 1 to 11c. The y-axis represents the percentage of differences in answering each question. It is visible that over 50% of answers did not differ at T1 and T2.
